# Comparative effectiveness and safety of tofacitinib vs. adalimumab in patients with rheumatoid arthritis: A systematic review and meta-analysis

**DOI:** 10.3389/fphar.2025.1524214

**Published:** 2025-06-09

**Authors:** Chunyan Zhu, Yibo Zheng, Zilu Wang, Guozong Chen, Yushi Li

**Affiliations:** ^1^ Department of Rheumatism and immunology, Dongying People’s Hospital (Dongying Hospital of Shandong Provincial Hospital Group), Dongying, Shandong, China; ^2^ Department of Rehabilitation Medicine, Dongying People’s Hospital (Dongying Hospital of Shandong Provincial Hospital Group), Dongying, Shandong, China

**Keywords:** tofacitinib, adalimumab, rheumatoid arthritis, meta-analysis, systematic review

## Abstract

**Objectives:**

To provide the latest systematic review and meta-analysis comparing the effectiveness and safety of tofacitinib and adalimumab in rheumatoid arthritis (RA) patients.

**Methods:**

A systematic search of PubMed, Embase, Web of Science, and Cochrane databases was conducted until April 2025. Randomized controlled trials and cohorts comparing tofacitinib and adalimumab in RA patients were included. Outcomes assessed were significant improvements in American College of Rheumatology (ACR) 20 improvement criteria, changes in visual analog scale (VAS) (global activity), disease activity score (DAS) 28-C-reactive protein (CRP), Health Assessment Questionnaire-Disability Index (HAQ-DI), and adverse events. Sensitivity analyses and subgroup analysis evaluated the robustness of results and heterogeneity. Data analysis was performed using Review Manager 5.4.1 and STATA 15.0.

**Results:**

Nine studies with 24,643 patients were analyzed. Tofacitinib showed superior effectiveness over adalimumab in ACR20 (risk ratio (RR): 1.28; 95% CI: 1.06, 1.55; P = 0.01), HAQ-DI (standardized mean difference (SMD): 0.20; 95% CI: 0.35, −0.05; P = 0.008), and VAS (SMD: 0.30; 95% CI: 0.56, −0.03; P = 0.03). No significant differences were found in adverse events (RR: 0.96; 95% CI: 0.89, 1.03; P = 0.22) or DSA28-CRP improvement (SMD: 0.02; 95% CI: 0.45, 0.02; P = 0.07). Sensitivity analyses confirmed stable outcomes for adverse events, HAQ-DI, and ACR20, but instability for VAS and DSA28-CRP. Subgroup analysis found that tofacitinib >5 mg twice daily was superior to ≤5 mg in terms of ACR20.

**Conclusion:**

Tofacitinib was more effective than adalimumab in improving ACR20, VAS, and HAQ-DI, with no significant differences in adverse events or DSA28-CRP improvement.

**Systematic Review Registration:**

https://www.crd.york.ac.uk/PROSPERO/.

## 1 Introduction

Rheumatoid arthritis (RA) is a chronic systemic autoimmune disease marked by persistent synovitis, pannus formation, and destruction of cartilage and bone (Zhu and Zhou, 2024; Vittecoq et al., 2024). The exact cause and mechanism in RA patients remain unknown. Clinically, it presents as symmetrical joint swelling, pain, and stiffness, potentially leading to joint deformities in advanced stages (Soriano et al., 2024). RA is prevalent worldwide, with varying rates across regions. Current early treatment primarily involves five classes of drugs: NSAIDs, csDMARDs, tsDMARDs, bDMARDs, and glucocorticoids (Qi et al., 2024; Han et al., 2024). Among them, adalimumab and tofacitinib are representative drugs of bDMARDs and tsDMARDs, respectively.

Adalimumab, the first fully humanized monoclonal antibody, binds specifically to TNF-α and effectively inhibits this cytokine (Zhang et al., 2024). The main risks of adalimumab include opportunistic infections and injection site reactions, and studies have shown that the incidence of serious infections is comparable to the underlying risk of rheumatoid arthritis (Smolen et al., 2024; Conde-Aranda et al., 2024; Lakhmiri et al., 2024). Tofacitinib, a first-generation oral drug, selectively inhibits JAK1 and JAK3, exerting less influence on JAK2 and TYK2 (Shih et al., 2024). Tofacitinib was first approved by the FDA for the treatment of RA in November 2012. Research indicates that tofacitinib is effective as a monotherapy or combined with DMARDs for RA treatment. The US FDA has approved tofacitinib for RA patients unresponsive to MTX (Fournier et al., 2024; Chang and Wang, 2024). In January 2017, after reviewing long-term safety and efficacy data, the European Medicines Agency proposed approving tofacitinib for RA treatment. In 2016, the FDA approved an extended-release tofacitinib formulation administered via an osmotic system for RA treatment (Boussaa et al., 2024; Adami et al., 2024). However, in February 2019, the FDA issued a safety alert regarding dose-dependent thrombosis and mortality risks with JAK inhibitors, which originated from the ORAL Surveillance study of tofacitinib in rheumatoid arthritis (RA) patients. Subsequent regulatory actions extended this boxed warning to the entire JAK inhibitor class (including baricitinib and upadacitinib), mandating restricted use in patients with cardiovascular risk factors regardless of specific indications (Kim et al., 2023; Fleischmann et al., 2017; van Vollenhoven et al., 2012; Fleischmann et al., 2012). A 24-week double-blind phase IIb study by Fleischmann et al. (2012) found that tofacitinib monotherapy (≥5 mg, twice daily) effectively treated active RA for over 24 weeks with manageable safety, compared to adalimumab (Fleischmann et al., 2012). Another study reported similar ACR20 outcomes for tofacitinib and adalimumab in RA treatment (Fleischmann et al., 2017).

Despite numerous clinical studies comparing tofacitinib and adalimumab for RA, evidence-based data confirming their relative advantages and disadvantages remains insufficient. This study aims to compare adalimumab and tofacitinib regarding clinical efficacy, disease activity, quality of life, and safety through systematic review and meta-analysis, providing clinicians with a reference for selecting treatments. The goal is to identify a regimen with rapid onset, strong efficacy, good safety, and low cost for RA patients.

## 2 Methods

### 2.1 Literature search

This meta-analysis followed the PRISMA 2020 guidelines (Page et al., 2021) and is registered in PROSPERO (CRD42024605000). A systematic literature search was conducted in PubMed, Embase, Web of Science, and Cochrane up to April 2025 for studies comparing adalimumab and tofacitinib in rheumatoid arthritis patients. The search terms used were “adalimumab,” “tofacitinib,” and “rheumatoid arthritis.” Detailed search strategies are provided in Supplementary Table S1. Additionally, we manually screened the reference lists of included studies. Two authors independently retrieved and assessed eligible articles, resolving any discrepancies through discussion.

### 2.2 Inclusion and exclusion criteria

Articles were included if they met the following criteria: P (Population): patients with rheumatoid arthritis; I (Intervention): tofacitinib; C (Comparison): adalimumab; O (Outcome): American College of Rheumatology (ACR) 20, change in patient-reported visual analog scale (VAS) (global activity), disease activity score (DAS) 28-C-reactive protein (CRP), change in Health Assessment Questionnaire-Disability Index (HAQ-DI), and adverse events, etc.; S (Study design): randomized controlled trials and cohort studies. Exclusion criteria included study protocols, unpublished or non-original studies (e.g., meeting abstracts, corrections, replies), single-arm studies, studies with insufficient data, and reviews.

### 2.3 Data abstraction

Data abstraction was independently performed by two authors, with discrepancies resolved by a third author. Abstracted data included: first author, publication year, research period, region, study design, registration number, population, intervention/exposure, control, sample size, age, gender, disease duration, follow-up, ACR20, VAS changes, DAS28-CRP changes, HAQ-DI changes, and adverse events. If data were incomplete, corresponding authors were contacted for additional information.

### 2.4 Quality evaluation

Risk of bias (RoB) tool for RCT quality evaluation followed the Cochrane Handbook for Systematic Reviews of Interventions 5.1.0, considering seven domains: randomization sequence generation, allocation concealment, participant and personnel blinding, outcome assessment blinding, incomplete outcome data, selective reporting, and other potential bias sources (Cumpston et al., 2019). Each aspect was rated as low, high, or unclear risk. Studies with more “low risk” evaluations were considered superior. Cohort study quality was assessed using the Newcastle-Ottawa Scale (NOS) (Wells et al., 2011), with scores of seven to nine indicating high quality (Kim et al., 2019). Two authors independently evaluated the quality of all included studies, resolving disagreements through discussion.

### 2.5 Statistical analysis

Data synthesis was conducted using Review Manager 5.4.1. Standardized mean difference (SMD) was applied for continuous data, and risk ratio (RR) for dichotomous data. We measured improvement in all continuous variables by calculating the change from baseline to the last follow-up visit. Each metric was reported with 95% CIs using a random-effects model. Heterogeneity across outcomes was assessed using the chi-squared (χ^2^) test (Cochran’s Q) and the inconsistency index (Higgins and Thompson, 2002). Substantial heterogeneity was defined as a χ^2^ P value below 0.1 or an I^2^ over 50%. For outcomes with more than two studies, sensitivity analysis was performed to assess the impact of individual studies on the overall results. In addition, subgroup analyses were performed to explore the stability of the results and potential sources of heterogeneity.

## 3 Results

### 3.1 Literature retrieval, study characteristics, and baseline

**FIGURE 1:**
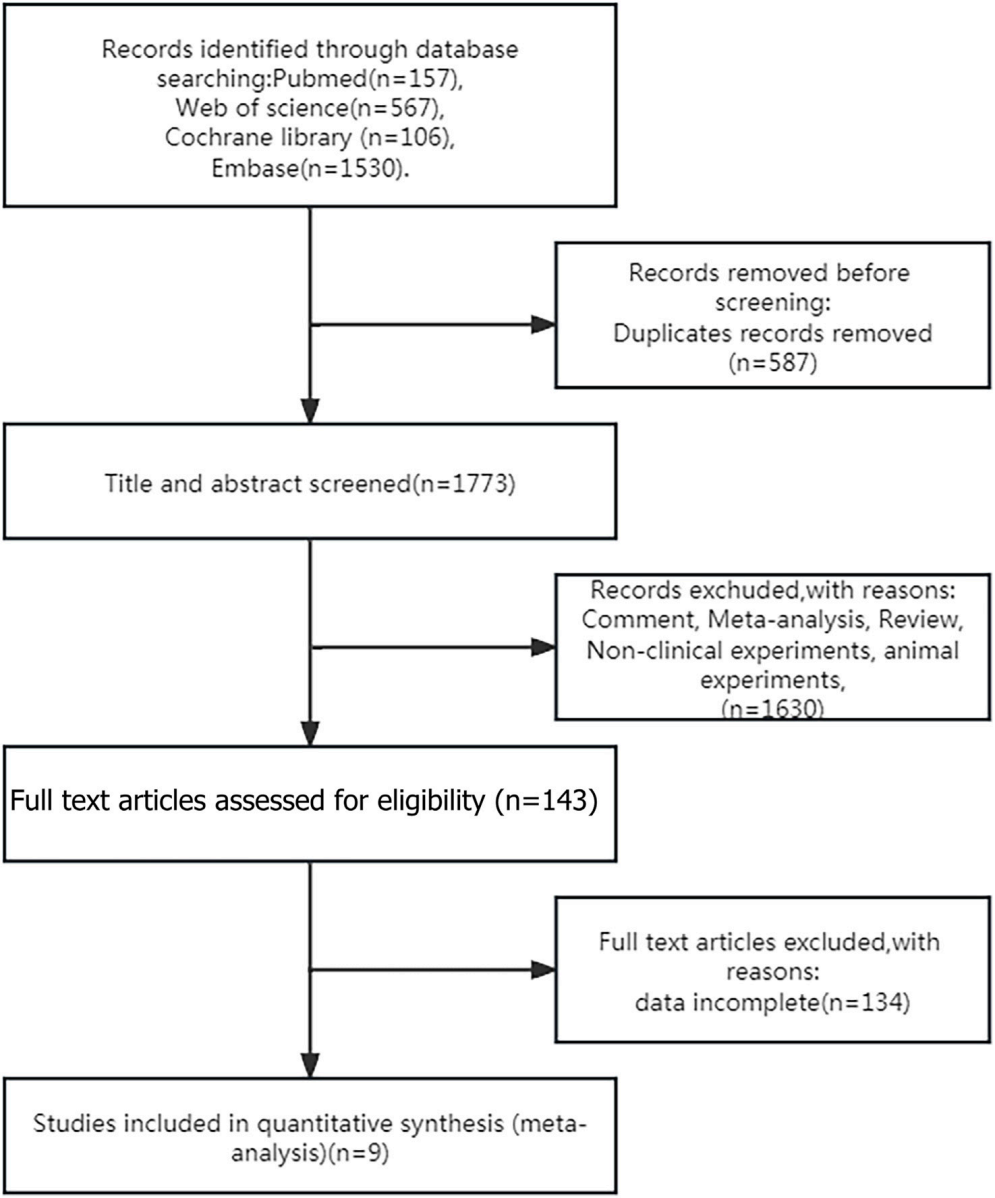
Flowchart of the systematic search and selection process.

**FIGURE 2:**
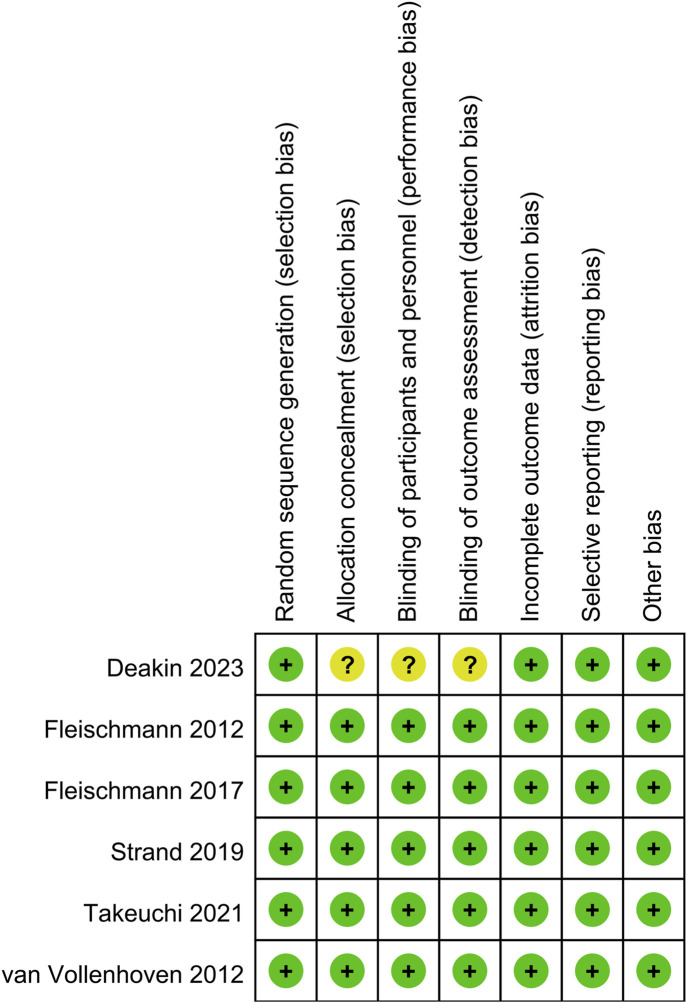
Details of the quality evaluation for included RCTs.

Figure 1 illustrates the flowchart of literature retrieval and selection. A total of 2,360 studies from PubMed (n = 157), Embase (n = 1,530), Web of Science (n = 567), and Cochrane (n = 106) were identified through a systematic search. After removing duplicates, 1,773 titles and abstracts were screened. Ultimately, nine studies (including 14 comparison groups) (Kim et al., 2023; Fleischmann et al., 2017; van Vollenhoven et al., 2012; Fleischmann et al., 2012; Deakin et al., 2023; Baker et al., 2023; Bergman et al., 2022; Takeuchi et al., 2021; Strand et al., 2019) involving 24,643 patients were included in the meta-analysis. Among them, six studies were RCTs and three studies were cohort studies. Table 1 summarizes the characteristics of each eligible study. Figure 2 shows the quality evaluation of all included RCTs. Quality assessments for included cohorts are provided in Supplementary Table S2.

**TABLE 1:** Characteristics of included studies.

Study	Study period	Country	Study design	Registration number	Population	Intervention/exposure	Control	Patients		Mean follow-up	Mean/median age		Male	
								tofacitinib	adalimumab		tofacitinib	adalimumab	tofacitinib	adalimumab
Kim 2023	2018–2020	Korea	Cohort	NCT03703817	Patients aged ≥19 years with patients who were taking tofacitinib or adalimumab for ≥6 months	tofacitinib	adalimumab	226	99	2 years	53.56	53.27	28	15
Deakin 2023	2015–2021	Australian	RCT	HC17799	Australian adults aged 18 years or older with RA in the Optimising Patient Outcomes in Australian Rheumatology (OPAL) data set	Tofacitinib (10 mg daily)	Adalimumab (40 mg every 14 days)	273	569	3 years	59	56	72	175
Baker 2023	2003–2019	United States	Cohort	NA	Patients aged 18 years or older with RA	tofacitinib	adalimumab	1565	13,326	1.8 years	57.6	52.7	271	3591
Bergman 2023	2018–2022	United States	Cohort	NA	Adults (aged C 18 years) with C 1 RA diagnosis	tofacitinib	adalimumab	1770	3732	12 months	52.1	49.7	301	897
Takeuchi 2021	NA	Multi-center	RCT	NCT02187055	Patients were ≥18 years of age with active RA per ACR/EULAR criteria [9], despite receiving MTX for ≥4 months before screening and at stable doses of 15–25 mg/week (<15 mg/week permitted only for safety reasons) for ≥6 weeks before baseline	Tofacitinib 5 mg BID + MTX	ADA 40 mg every other week + MTX	311	314	12 months	NA	NA	NA	NA
Strand 2019	NA	Multi-center	RCT	NCT02187055	≥18 years of age and met ACR/European League Against Rheumatism classification criteria for active RA.	Oral tofacitinib was dosed at 5 mg two times per day + MTX	Subcutaneous ADA was dosed at 40 mg Q2W + MTX	376	386	NA	NA	NA	NA	NA
Fleischmann 2017	2014–2015	Multi-center	RCT	NCT02187055	aged 18 years or older who met the 2010 ACR and EULAR classification criteria for rheumatoid arthritis20	tofacitinib 5 mg BID + MTX	ADA 40 mg every other week + MTX	376	386	12 months	50	50	65	66
van Vollenhoven 2012a	2009–2011	Multi-center	RCT	NCT00853385	18 years of age or older and had received a diagnosis of active rheumatoid arthritis	5 mg of tofacitinib twice daily	40 mg of adalimumab administered by subcutaneous injection once every 2 weeks	204	204	6 months	53	52.5	30	42
van Vollenhoven 2012b	2009–2011	Multi-center	RCT	NCT00853385	18 years of age or older and had received a diagnosis of active rheumatoid arthritis	10 mg of tofacitinib twice daily	40 mg of adalimumab administered by subcutaneous injection once every 2 weeks	201	204	6 months	52.9	52.5	33	42
Fleischmann 2012a	NA	Multi-center	RCT	NCT00550446	≥18 years of age, had a diagnosis of RA for ≥6 months	1 mg twice a day	Injected subcutaneously at 40 mg once every other week	54	53	12 weeks	55	54	8	8
Fleischmann 2012b	NA	Multi-center	RCT	NCT00550446	≥18 years of age, had a diagnosis of RA for ≥6 months	3 mg twice a day	Injected subcutaneously at 40 mg once every other week	51	53	12 weeks	53	54	7	8
Fleischmann 2012c	NA	Multi-center	RCT	NCT00550446	≥18 years of age, had a diagnosis of RA for ≥6 months	5 mg twice a day	Injected subcutaneously at 40 mg once every other week	49	53	12 weeks	54	54	6	8
Fleischmann 2012d	NA	Multi-center	RCT	NCT00550446	≥18 years of age, had a diagnosis of RA for ≥6 months	10 mg twice a day	Injected subcutaneously at 40 mg once every other week	61	53	12 weeks	52	54	8	8
Fleischmann 2012e	NA	Multi-center	RCT	NCT00550446	≥18 years of age, had a diagnosis of RA for ≥6 months	15 mg twice a day	Injected subcutaneously at 40 mg once every other week	57	53	12 weeks	53	54	7	8

### 3.2 ACR20

**FIGURE 3:**
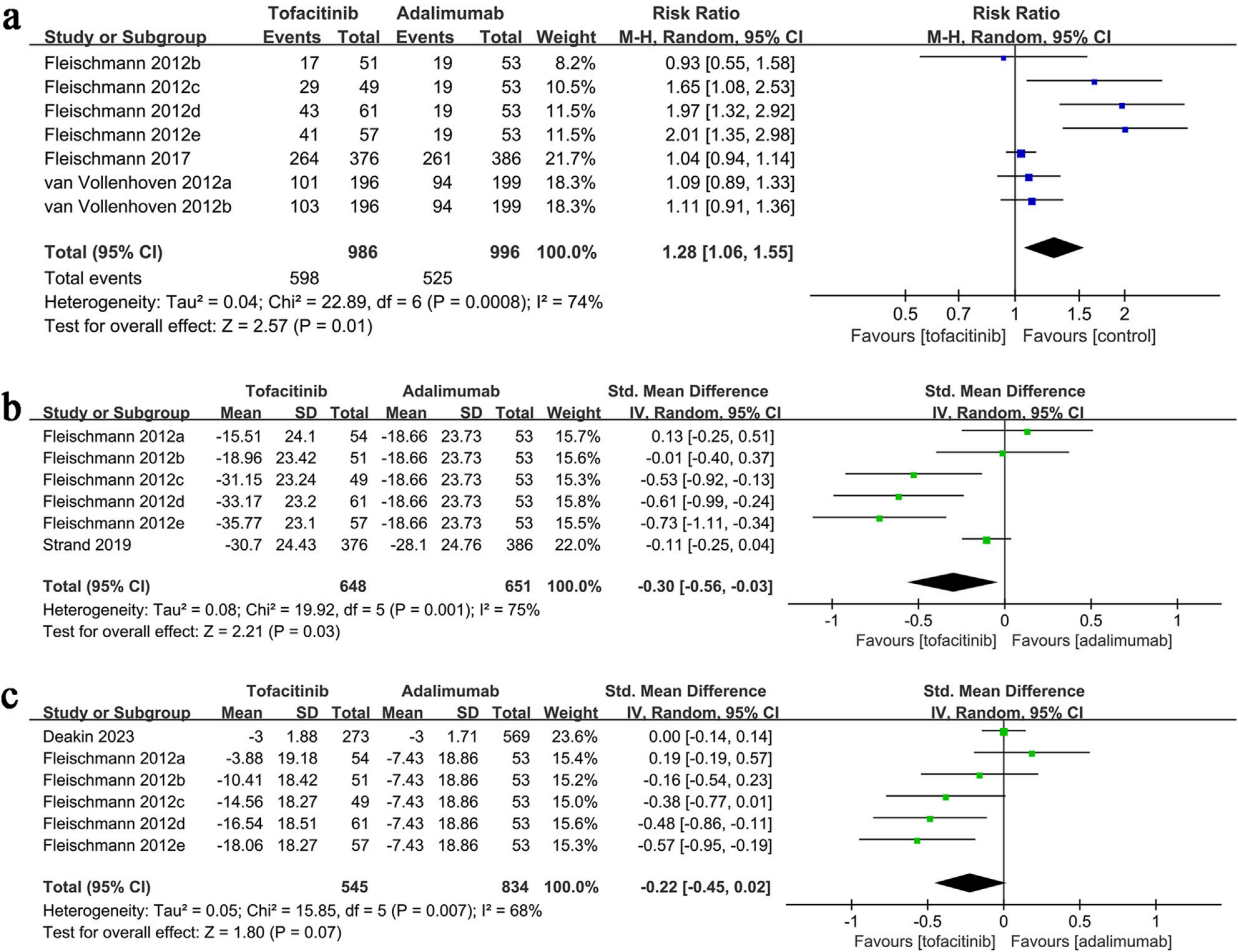
Forest plots of **(a)** ACR20, **(b)** change in VAS, **(c)** change in DAS28-CRP.

ACR20 results were synthesized from three studies (7 comparison groups) involving 1,982 patients. Meta-analysis showed a significantly higher ACR20 in the tofacitinib group (RR: 1.28; 95% CI: 1.06, 1.55; P = 0.01) with substantial heterogeneity (I^2^ = 74%, P = 0.0008) (Figure 3a).

### 3.3 Change in VAS

VAS change data synthesis was performed in two studies (6 comparison groups) involving 1,299 patients. Meta-analysis showed a significantly greater reduction in VAS in the tofacitinib group (SMD: 0.30; 95% CI: 0.56, −0.03; P = 0.03) with substantial heterogeneity (I^2^ = 75%, P = 0.001) (Figure 3b).

### 3.4 Change in DAS28-CRP

Results of change in DAS28-CRP were synthesized from two studies (including six comparison groups) including 1,379 patients. Meta-analysis revealed a similar change in DAS28-CRP in the tofacitinib and adalimumab group (SMD: 0.02; 95% CI: 0.45, 0.02; *P* = 0.07) with a significant heterogeneity (*I* ^2^ = 68%, *P* = 0.007) (Figure 3c).

### 3.5 Change in HAQ-DI

**FIGURE 4:**
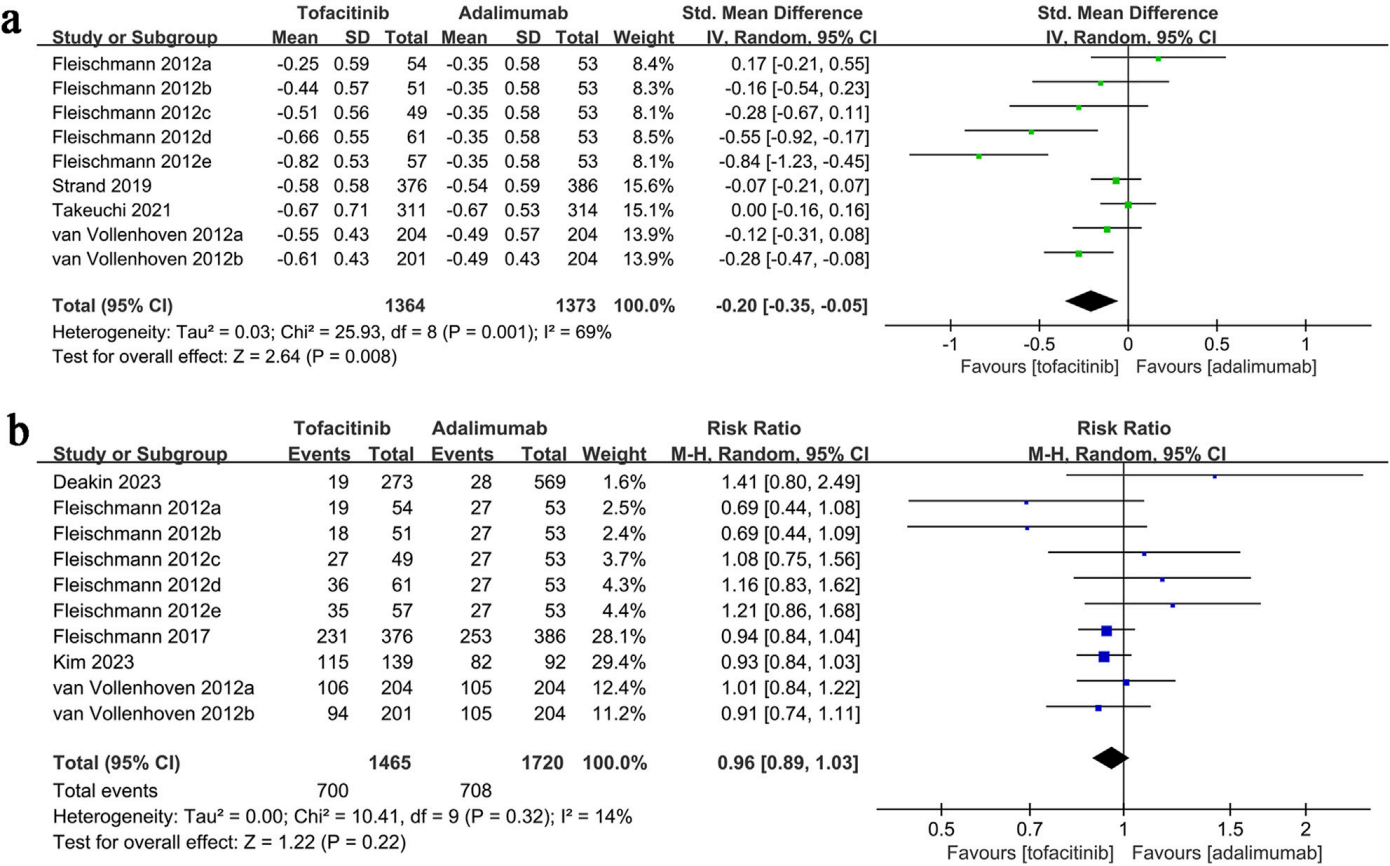
Forest plots of **(a)** change in HAQ-DI, **(b)** adverse events.

HAQ-DI change data synthesis was performed in four studies (9 comparison groups) involving 2,737 patients. Meta-analysis showed a significantly greater reduction in HAQ-DI in the tofacitinib group (SMD: 0.20; 95% CI: 0.35, −0.05; P = 0.008) with substantial heterogeneity (I^2^ = 69%, P = 0.001) (Figure 4a).

### 3.6 Adverse events

Adverse events data were synthesized from five studies (10 comparison groups) involving 3,185 patients. Meta-analysis showed a similar adverse event rate between the tofacitinib and adalimumab groups (RR: 0.96; 95% CI: 0.89, 1.03; P = 0.22) with no significant heterogeneity (I^2^ = 14%, P = 0.32) (Figure 4b).

### 3.7 Subgroup analysis

**FIGURE 5:**
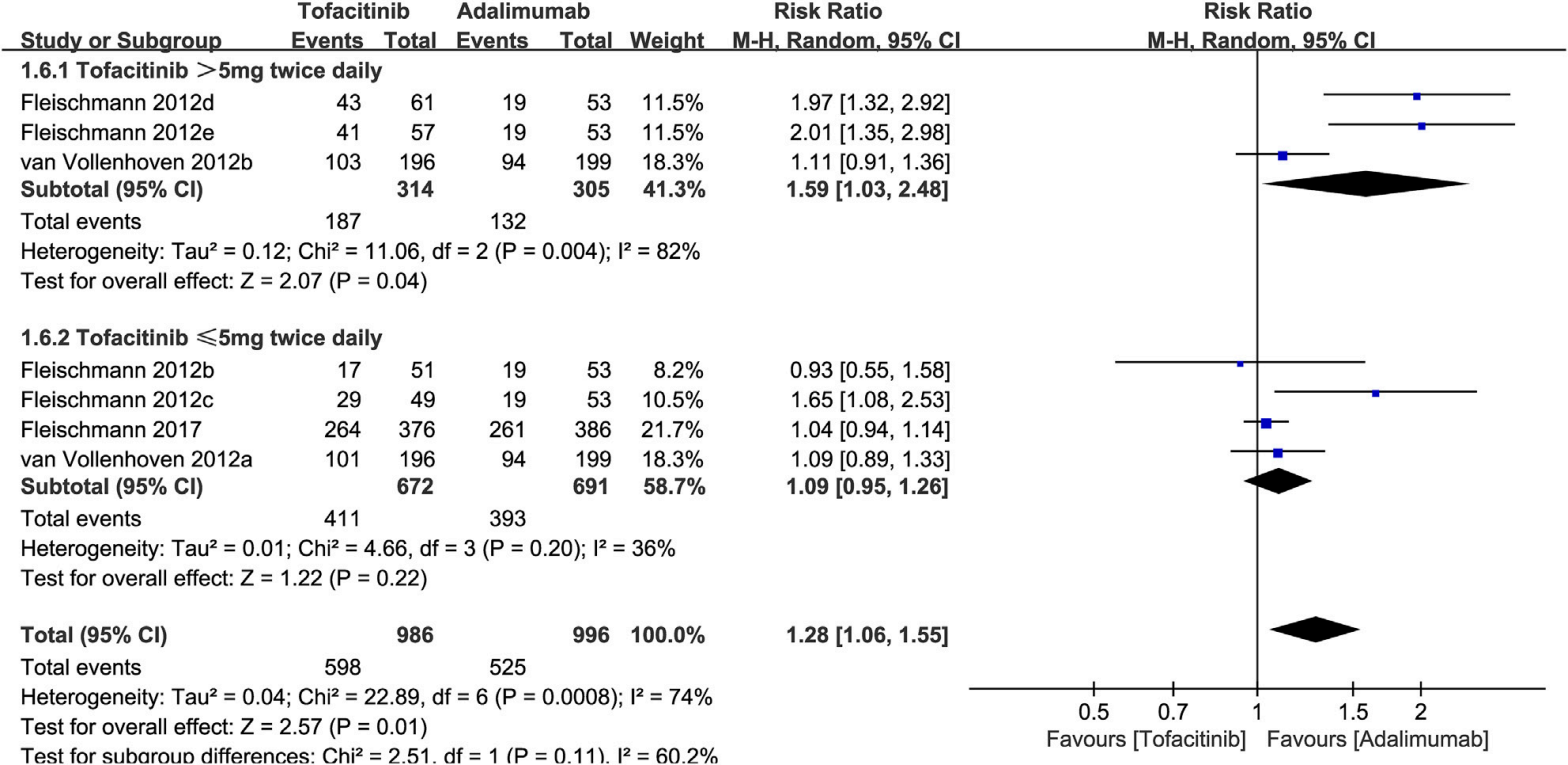
Subgroup analysis of ACR20 based on the dose of tofacitinib.

To explore the effect of tofacitinib dose on efficacy, we performed a subgroup analysis of ACR20. The results showed that when the dose was >5 mg twice a day, the ACR20 of tofacitinib was significantly better than that of adalimumab (RR: 1.59; 95% CI: 1.03, 2.48; P = 0.04). However, in the subgroup with a dose of ≤5 mg twice a day, tofacitinib and adalimumab had similar ACR20 (RR: 1.09; 95% CI: 0.95, 1.26; P = 0.22) (Figure 5).

### 3.8 Sensitivity analysis

**FIGURE 6:**
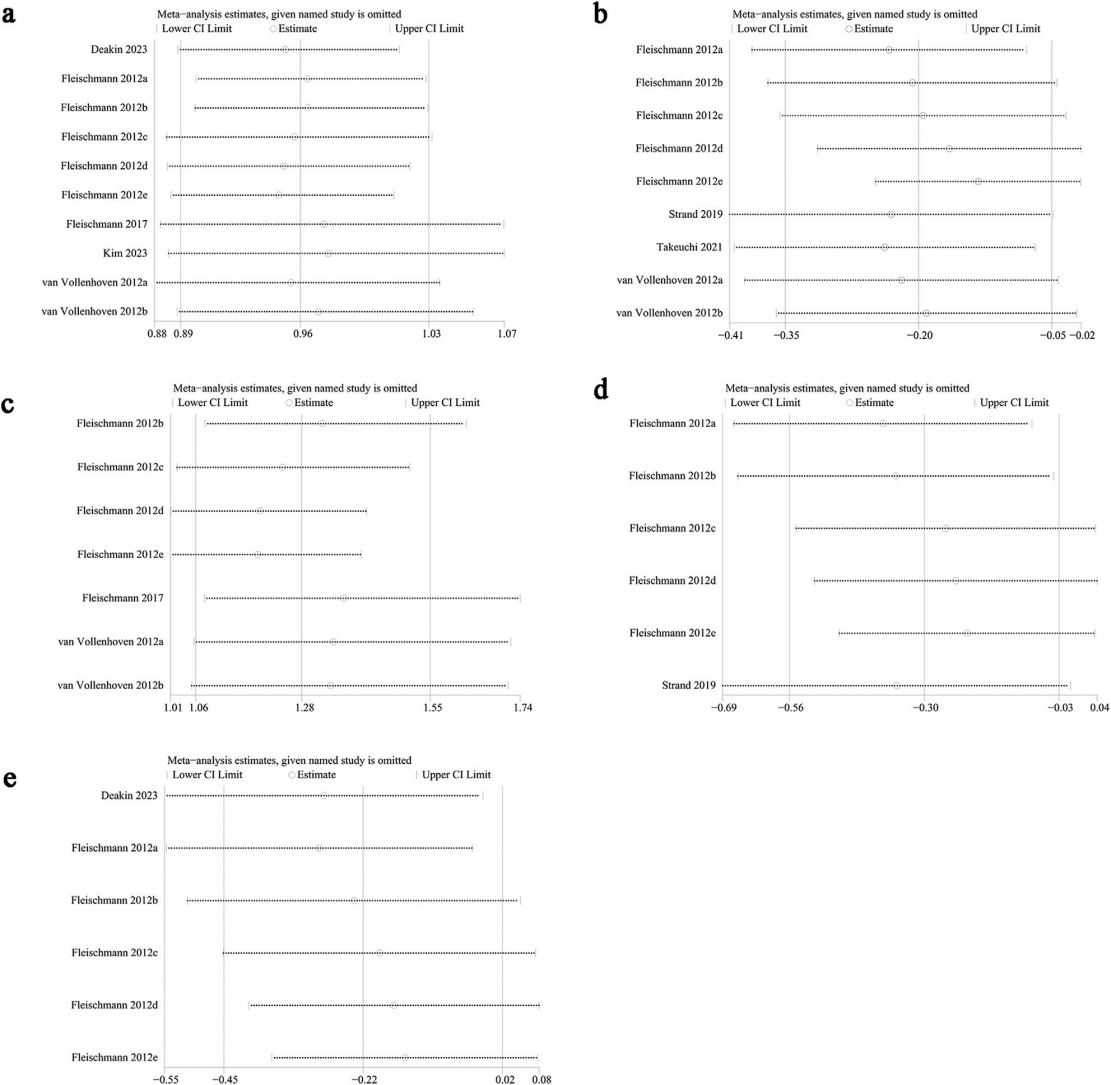
Sensitivity analysis of **(a)** adverse events, **(b)** change in HAQ-DI, **(c)** ACR20, **(d)** change in VAS, **(e)** change in DAS28-CRP.

Sensitivity analyses were conducted for ACR20, VAS, DAS28-CRP, HAQ-DI, and adverse events to evaluate the effect of each study on overall outcomes by sequentially excluding eligible studies. The analyses showed stable outcomes after excluding each study for adverse events (Figure 6a), HAQ-DI (Figure 6b), and ACR20 (Figure 6c). However, significant instability was found for VAS (Figure 6d) and DAS28-CRP (Figure 6e). For HAQ-DI, excluding Fleischmann 2012e reduced heterogeneity from 69% to 47%, indicating that this study contributed to the significant heterogeneity. Heterogeneity in other outcomes was not linked to a specific study.

## 4 Discussion

Current treatments for RA primarily consist of drug therapies, including NSAIDs, csDMARDs, tsDMARDs, bDMARDs, and glucocorticoids. TNF-α is a key inflammatory factor in RA pathogenesis, regulating osteoclast production and inhibiting osteoblast differentiation, which disrupts the balance between osteoclasts and osteoblasts, leading to bone and joint damage (Bertolini et al., 1986). Research indicates that patients with high TNF-α expression have a significantly higher risk of erosive arthritis (Laverdière et al., 2015). Adalimumab is the first fully humanized monoclonal antibody that specifically targets TNF-α. Tofacitinib, a first-generation oral drug, selectively inhibits JAK1 and JAK3, with minimal effect on JAK2 and TYK2 (Clark et al., 2014). Studies show that tofacitinib is effective as monotherapy or in combination with DMARDs for RA (Chang and Wang, 2024).

Nine studies were included in this meta-analysis. Results showed that tofacitinib significantly outperformed adalimumab in ACR20, HAQ-DI, and VAS improvements. No significant difference was observed in adverse events or DAS28-CRP improvements between the two groups. ACR20 was the primary efficacy indicator in this analysis. Van Vollenhoven et al. (van Vollenhoven et al., 2012) found that at month 6, similar proportions of patients on tofacitinib and adalimumab achieved ACR20, both exceeding placebo. However, this meta-analysis found significantly higher ACR20 in the tofacitinib group than in the adalimumab group. This discrepancy was largely due to the inclusion of Fleischmann et al.'s (Fleischmann et al., 2012) dose-response study. At doses of ≥5 mg (twice daily), tofacitinib’s efficacy was significantly superior to adalimumab 40 mg (once every 2 weeks). van Vollenhoven et al. (2012) also observed a trend suggesting tofacitinib’s superiority over adalimumab in ACR20, though this was not statistically significant. Thus, we believe that when the tofacitinib dose exceeds 5 mg (twice daily), its ACR20 is significantly higher than that of the conventional adalimumab dose. However, the long-term efficacy of both drugs in treating rheumatoid arthritis requires further investigation in future clinical studies with extended follow-up durations and additional time points.

Although ACR20 is a commonly used indicator in RA clinical trials, its sensitivity to functional improvement is limited. In addition, the ACR20 standard currently only applies to the US FDA regulatory system and has not been adopted by agencies such as MHRA and EMA. This study also included patient-reported outcomes such as HAQ-DI to more comprehensively evaluate the impact of treatment on quality of life. Joint pain, swelling, and functional limitation are key clinical manifestations of RA (Skyrme et al., 2024; Looijen et al., 2024; Frazzei et al., 2024). This study found tofacitinib significantly superior to adalimumab in improving pain scores. However, Strand et al. (2019) did not observe a significant advantage of tofacitinib in improving VAS, possibly due to sample size limitations. In assessing quality of life, this meta-analysis found tofacitinib significantly superior to adalimumab in improving HAQ-DI. However, Strand et al. (2016) reported similar HAQ-DI improvements in both treatment groups at all time points, differing from the results of this study. Differences in race, disease course, and body mass index may explain varying patient responses to treatment. Additionally, no significant difference was observed between tofacitinib and adalimumab in improving DAS28-CRP, suggesting similar effects in reducing RA activity and delaying bone destruction. Tofacitinib’s primary advantage lies in improving quality of life and symptom relief. The long-term prognostic differences between these two drugs in rheumatoid arthritis treatment require further study.

This study found comparable incidence rates of adverse events between the two groups in the safety evaluation. The safety of these two drugs remains controversial in current reports. While some studies align with this article (Fleischmann et al., 2017), van Vollenhoven et al. (2012) reported a higher probability of serious adverse events with tofacitinib than adalimumab within the first 3 months of treatment. Given the sample size limitations, further research is needed to confirm this finding.

This meta-analysis has several limitations. First, the study included a small number of retrospective cohort studies, inherent in clinical research. A major limitation of retrospective studies is the potential for confounding factors and bias. In addition, not all included studies were international multicenter studies. Some studies only included populations from a single country or region, resulting in a certain degree of selective bias. Besides, due to insufficient data, we were unable to analyze the differences between tofacitinib and adalimumab in important clinical indicators such as ACR50, which needs to be confirmed by further studies. In addition, merging different types of studies may produce “mixed effects” that are difficult to explain. For example, the effect size of RCTs is based on strictly controlled intervention conditions, while the effect size of cohort studies is easily interfered by real-world confounding factors (such as lifestyle and comorbidities). The combined results of the two may not be pure causal effects or true exposure associations. At the same time, the non-compressibility of OR values ​​may cause the combined effect to deviate from the true value. Furthermore, if the patient stops treatment prematurely, the results at the last follow-up may not reflect the sustained treatment effect, which may affect the overall efficacy analysis of the drug to some extent. Finally, due to insufficient data, the economic costs of tofacitinib and adalimumab could not be analyzed, requiring further investigation. Due to the limited number of studies, detailed subgroup and dose-response analyses could not be performed, leaving the effects of factors such as population, intervention duration, follow-up time, and drug dose unconfirmed. Although significant heterogeneity was present in this meta-analysis, sensitivity analysis did not fully identify its source, warranting caution when interpreting the results. Despite these limitations, this is the latest meta-analysis comparing the efficacy and safety of tofacitinib and adalimumab. The findings provide clinicians with the most up-to-date, comprehensive evidence-based reference for selecting treatments, aiming to identify options with rapid onset, strong efficacy, safety, and affordability for RA patients.

## 5 Conclusion

This meta-analysis found tofacitinib significantly superior to adalimumab in improving ACR20, VAS, and HAQ-DI, with no significant difference in adverse event rates or DAS28-CRP improvement between the two. Given the limitations of ACR20, clinical decision making needs to comprehensively consider patient-reported outcomes, long-term safety, and individualized treatment goals. Given the potential heterogeneity, small sample size, and lack of subgroup analysis, larger multicenter prospective studies are needed to confirm the advantages and disadvantages of tofacitinib and adalimumab in RA treatment.

## Data Availability

The original contributions presented in the study are included in the article/Supplementary Material, further inquiries can be directed to the corresponding author.
