# Supplementary material for: Comparative effectiveness and safety of tofacitinib vs. adalimumab in patients with rheumatoid arthritis: A systematic review and meta-analysis
Source: Front Pharmacol. 2025 Jun 9;16:1524214. doi: 10.3389/fphar.2025.1524214 (PMC12183229; doi:10.3389/fphar.2025.1524214)
Supplement: Supplementary file 1 [file DataSheet1.docx]

**Supplementary materials**

TableS1 Literature Search Strategy

Pubmed-157

((("Adalimumab"[Mesh]) OR (((((((Humira) OR (Adalimumab-adbm)) OR (Amjevita)) OR (Adalimumab-atto)) OR (Cyltezo)) OR (D2E7 Antibody)) OR (Antibody, D2E7))) AND (("tofacitinib" [Supplementary Concept]) OR (((((((((tasocitinib) OR (tofacitinib)) OR (tofacitinib citrate)) OR (Xeljanz)) OR (CP 690,550)) OR (CP690550)) OR (CP-690550)) OR (CP 690550)) OR (CP-690,550)))) AND (("Arthritis, Rheumatoid"[Mesh]) OR (Rheumatoid Arthritis))

Embase-1530


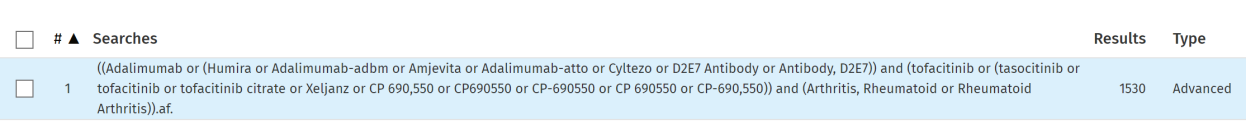


Cochrane-106


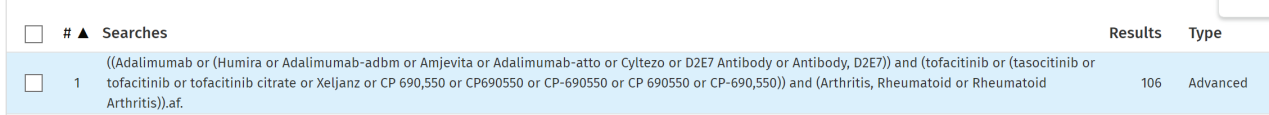


WOS-567

(((Adalimumab) OR (((((((Humira) OR (Adalimumab-adbm)) OR (Amjevita)) OR (Adalimumab-atto)) OR (Cyltezo)) OR (D2E7 Antibody)) OR (Antibody, D2E7))) AND ((tofacitinib) OR (((((((((tasocitinib) OR (tofacitinib)) OR (tofacitinib citrate)) OR (Xeljanz)) OR (CP 690,550)) OR (CP690550)) OR (CP-690550)) OR (CP 690550)) OR (CP-690,550)))) AND ((Arthritis, Rheumatoid) OR (Rheumatoid Arthritis)) (Topic) and Preprint Citation Index (Exclude – Database)

| Supplementary Table S2. Quality evaluation of the eligible studies with Newcastle–Ottawa scale. | | | | | | | | | |
| --- | --- | --- | --- | --- | --- | --- | --- | --- | --- |
| Study | Selection | | | | Comparability | | Outcome | | |
|  | Representative-ness | Selection of  non-exposed | Ascertainment  of exposure | Outcome not present at start | Comparability on most important factors | Comparability on other risk factors | Assessment of outcome | Long enough follow-up (median≥1 year) | Adequacy  (completeness) of follow-up |
| Kim et al. | * | * | * | * | * | - | * | - | * |
| Gharaibeh et al. | * | * | * | * | - | - | * | * | * |
| Deakin, et al. | * | * | * | * | - | - | * | - | * |
| Baker et al. | * | * | * | * | * | - | * | * | * |
| Bergman et al. | * | * | * | * | - | - | * | * | * |
| Chen et al. | * | * | * | * | - | - | * | * | * |
| *indicates criterion met; - indicates significant of criterion not met. | | | | | | | | | |
